# A Fusion between Domains of the Human Bone Morphogenetic Protein-2 and Maize 27 kD γ-Zein Accumulates to High Levels in the Endoplasmic Reticulum without Forming Protein Bodies in Transgenic Tobacco

**DOI:** 10.3389/fpls.2016.00358

**Published:** 2016-03-24

**Authors:** Valentina Ceresoli, Davide Mainieri, Massimo Del Fabbro, Roberto Weinstein, Emanuela Pedrazzini

**Affiliations:** ^1^Istituto di Biologia e Biotecnologia Agraria, Consiglio Nazionale Delle RicercheMilano, Italy; ^2^Dipartimento Scienze Biomediche, Chirurgiche e Odontoiatriche, Università Degli Studi di MilanoMilano, Italy; ^3^IRCCS Istituto Ortopedico GaleazziMilano, Italy

**Keywords:** bone morphogenetic protein 2, endoplasmic reticulum, protein accumulation, protein bodies, γ-zein, plant factories

## Abstract

Human Bone Morphogenetic Protein-2 (hBMP2) is an osteoinductive agent physiologically involved in bone remodeling processes. A commercialized recombinant hBMP2 produced in mammalian cell lines is available in different clinical applications where bone regeneration is needed, but widespread use has been hindered due to an unfavorable cost/effective ratio. Protein bodies are very large insoluble protein polymers that originate within the endoplasmic reticulum by prolamine accumulation during the cereal seed development. The N-terminal domain of the maize prolamin 27 kD γ-zein is able to promote protein body biogenesis when fused to other proteins. To produce high yield of recombinant hBMP2 active domain (ad) in stably transformed tobacco plants we have fused it to the γ-zein domain. We show that this zein-hBMP2ad fusion is retained in the endoplasmic reticulum without forming insoluble protein bodies. The accumulation levels are above 1% of total soluble leaf proteins, indicating that it could be a rapid and suitable strategy to produce hBMP2ad at affordable costs.

## Introduction

Throughout the adult life, as well as in the healing process following injuries, bone tissue is subjected to cycles of absorption and formation during the physiological modeling and remodeling. The former allows to shaping bone structure according to the loading situation, adapting cortical, and trabecular architecture to the functional needs, while the latter consists of the renewal of bone composition without changing the bone architecture. However, a massive bone regeneration may be required in pathological conditions such as in the presence of extensive bony defects caused by trauma, bone cancer, infection and necrosis, or because the remodeling process is compromised, such as in osteoporosis (Giannoudis et al., 2005).

The availability of Bone Morphogenetic Proteins (hBMPs), the osteoinductive agents in bone, as an adjunct to surgical procedures involving bone reconstruction, might reduce or avoid the need for complex and demanding surgeries, preventing major costs, and morbidity related to autograft harvesting (Garrison et al., 2007; Lo et al., 2012). Among the 20 members of hBMPs, which belong to TGF beta superfamily, only hBMP-2, -4, -6, -7, -9, and -14 have shown promising osteoinductive properties in different injuries (e.g., long bone fracture non-unions, spinal fusion, and maxillofacial bone defects; Even et al., 2012; Lo et al., 2012; Carreira et al., 2014). hBMP2 and hBMP7 are the most characterized factors and are able to strongly induce osteoblast differentiation in different tissues (Marie et al., 2002). In 2007, an extensive survey on the cost-effectiveness of the use of recombinant hBMPs (rhBMPs) in orthopedy concluded that there is lack of evidence about the clinical effectiveness and that their use would not be cost-effective unless the price is significantly reduced, except for severe cases (Garrison et al., 2007). Over the past 10 years, evidence about the clinical efficacy of rhBMP2 has been provided by sponsored clinical studies (Burks and Nair, 2010; Kim et al., 2015). Based on preliminary clinical results in oral, maxillofacial and orthopedic surgeries, rhBMP2 is as effective as the conventional grafting according to clinical and histomorphometric parameters, and in some cases it may accelerate bone healing (Kelly et al., 2015; Lin et al., 2015; Poon et al., 2016). Moreover, rhBMP2 may decrease morbidity and improve other patient-associated outcomes, with no signs of rejection or infection (Alt et al., 2015; Lin et al., 2015; Poon et al., 2016). On the other hand, while complications and adverse events were rarely reported using rhBMPs for their specific clinical indications, pitfalls have been observed with off-label use of rhBMP2, leading to a number of adverse effects (Poon et al., 2016). Finally, some authors have still expressed concern regarding the actual effectiveness and safety of rhBMPs, highlighting the risk of potential serious complications, as reported by non-sponsored studies (Even et al., 2012).

Despite the promising clinical results and the improvements in the production procedures of recent years, the widespread therapeutic use of rhBMPs has been hindered until now, both because of the FDA limitations on their applications, and the failure to obtain high amount of pure and biologically active protein at an affordable price (Harada et al., 2012; Carreira et al., 2014).

hBMP2 is synthesized as an inactive dimer that undergoes major posttranslational modifications to become biologically active. Each of the BMP-2 monomers (405 AA) contains a cysteine-knot which is based on six cysteine residues (C296, C325, C329, C361, C393, C395) forming three intra-chain disulfide bridges (Scheufler et al., 1999). The cystine-knot scaffold confers rigidity to the structure and it is necessary to stabilize the whole protein. The hBMP2 dimers are formed by a single inter-chain disulfide bond which engages the unpaired C360 of each monomer (Scheufler et al., 1999). The key post-translational modification is the endoproteolytic cleavage by proprotein convertase downstream the R282 in the KREKR consensus site that releases the mature homodimeric hBMP2 active domain (Heng et al., 2010).

Several attempts to express recombinant active hBMPs in different heterologous systems (such as *Escherichia coli, P. pastoris*, Baculovirus/insect cells, and mammalian cells) have been made (Colin et al., 2008). However, the production has encountered difficulties, especially due to the impairment of protein folding, different grade or absence of post-translational modifications and low protein stability (Hazama et al., 1995; Pulkki et al., 2011; Park et al., 2014.). In 2002 the first recombinant hBMP2 obtained from CHO cell lines was approved by the FDA (FDA approval number: P000054). Although mammalian cells are disadvantageous in terms of complexity and cost, the recombinant hBMP2 (rhBMP-2) and rhBMP-7 by Medtronic are so far the only ones to be marketed.

During the last two decades, plants have emerged as one of the most promising general production platforms for biologics, and nowadays plant-based expression systems are accepted as robust, scalable and cost-efficient platforms for the production of recombinant proteins of pharmaceutical interest, including antibodies, blood substitutes, vaccines, and growth factors (Ma et al., 2003; Fischer et al., 2004; Stoger et al., 2005; Melnik and Stoger, 2013; Sack et al., 2015).

A previous attempt to produce rhBMP2 in tobacco plants has been made (Suo et al., 2006). The level of accumulation of a recombinant protein in transgenic plants is protein specific and strongly influenced by the subcellular compartment of destination (Vitale and Pedrazzini, 2005); thus, search for the best subcellular compartment for the protein of interest represents a major issue in the effort to maximize production (Vitale and Pedrazzini, 2005; Hofbauer and Stoger, 2013). Several targeting strategies have been developed to improve protein accumulation in plant cells and one of the most promising is to exploit the seed storage protein determinants deputed to the formation of large oligomers that accumulate in the endoplasmic reticulum of maize endosperm, resulting in protein body (PB) biogenesis. The 27 kD γ-zein (hereafter zein) is a maize storage protein belonging to the prolamin class and is able to induce PB biogenesis even when expressed in vegetative tissues of transgenic plants (Shewry et al., 1995; Vitale and Ceriotti, 2004). Its N-terminal domain, characterized by eight repeats of the hexapeptide VHLPPP and seven cysteine residues is necessary for retention and deposition into the ER (Geli et al., 1994; Mainieri et al., 2014). The cysteine residues form inter-chain disulphide bonds, leading to assembly into the very large PBs. These polymers are therefore insoluble unless treated with reducing agents (Mainieri et al., 2004, 2014; Pompa and Vitale, 2006). This domain has been used to allow accumulation of recombinant fusion proteins both in transgenic plants and in mammalian cells (Mainieri et al., 2004; Llop-Tous et al., 2011).

In order to investigate new cost-effective approaches to express the hBMP2 in tobacco transgenic plants, we produced a chimeric protein (named zein-hBMP2ad), consisting of the C-terminal active domain of hBMP2 fused to the N-terminal domain of 27 kD γ-zein. We show that the prolamin domain promotes higher accumulation level of hBMP2ad compared to the native hBMP2 (hBMP2nat). zein-hBMP2ad assembly, post-translational modifications and ability to induced PB biogenesis were analyzed.

## Materials and methods

### Plasmid construction

**Figure 1:**
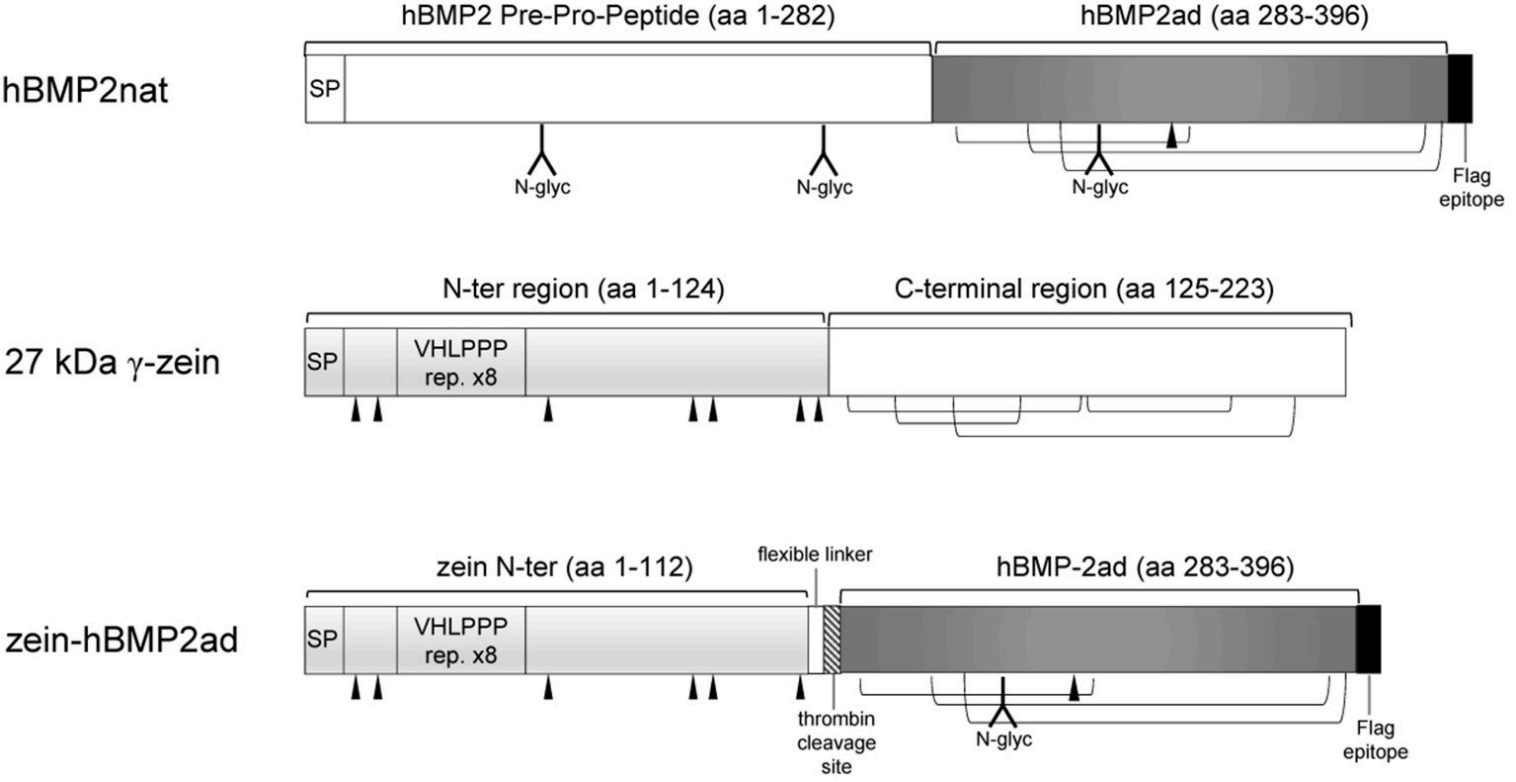
**Schematic representation of hBMP2nat, 27 kD γ-zein, and zein-hBMP2ad fusion**. Dark gray box: human BMP2 active domain (ad); light gray box: 27 kD γ-zein N-terminal domain; hatched box: thrombin consensus cleavage site; black triangles: cysteine residues involved in interchain-disulfide bridges. N-glycosylated sites in hBMP2 are indicated. Intrachain disulphide bridges in hBMP2ad and in the C-terminal region of 27 kD γ-zein are indicated. SP: Signal Peptide. Expected Molecular Mass: hBMP2nat, 45.8 kD; hBMP2ad, 14 kD; 27 kD γzein, 23.7 kD; zein-hBMP2ad, 27.4 kD.

The constructs used in this study are shown in Figure 1, and were prepared according to standard molecular techniques (Sambrook et al., 1989).

A single chimeric construct including the N-Terminal of 27 kDa γ-zein, a DNA linker, a Thrombin cleavage site, the complete coding sequence of human BMP2 and a FLAG epitope was purchased from GeneCust (GeneCust Europe, Laboratoire de Biotechnologie du Luxembourg S.A.) in a pUC57 vector in order to obtain the two zein-hBMP2ad and hBMP2nat constructs with specific subcloning experiments.

The chimeric gene zein-hBMP2ad was obtained by double digestion with XbaI and PstI and the insertion of the excised fragment in a pDHA vector. To reach the final sequence this plasmid was digested with AflII in order to excise the signal peptide and the pro-peptide of hBMP2. The resulting construct was used for transient expression in tobacco protoplasts. Full-length hBMP2 (hBMP2nat) was obtained by digestion with SalI and PstI. The fragment was then subcloned in a pDHA vector and used for transient expression in tobacco protoplasts.

For stable plant transformation, the EcoRI fragments excised from the two pDHA plasmids, containing the expression cassettes, were subcloned into EcoRI-linearized pGreenII 0179 (http://www.pgreen.ac.uk, John Innes Centre, Norwich, Norfolk, UK) that carries the hygromycin selectable marker gene. These constructs, called pGreen-ER and pGreen-N, were used to transform *A. tumefaciens* strain GV3101 by electroporation, as described by Shen and Forde (1989).

### Antibodies and recombinant protein

Rabbit polyclonal anti-FLAG (1:2000 Sigma Aldrich, St. Louis, MO, USA); rabbit polyclonal anti-tobacco BiP (1:10,000 dilution, Pedrazzini et al., 1997); rabbit polyclonal anti-endoplasmin/GRP94 (1:1000 dilution; Klein et al., 2006); goat anti-rabbit IgG-peroxidase conjugate (1:16,000, Pierce Biotechnology (Rockford, IL, USA). FLAG-Bap recombinant fusion protein (Sigma Aldrich, St. Louis, MO, USA).

### Plant material

Nicotiana tabacum plants (cv. Petit Havana SR1) as well as transgenics were cultured in sterile conditions on Murashige and Skoog (MS) basal salt medium (Duchefa Biochemie, Haarlem, the Netherlands) containing 0.8% plant agar (Duchefa Biochemie) or in soil in a growth room at 25°C with a 16-h/8-h light/dark cycle.

### *A. tumefaciens*-mediated transformation

Young tobacco leaves were excised from axenically grown wild-type plants and cut into about 1 cm2 leaf discs. The discs were placed for 5 min in a culture of *A. tumefaciens* carrying the plasmid of interest, and then incubated at 25°C in 16 h of light on MS salts medium (Duchefa Biochemie), containing 3% sucrose and 0.8% phyto agar, underside down. After 2 days, the leaf discs were transferred to a shoot generation medium (1/2 MS salts supplemented with 30 g/l sucrose, 0.1 mg/l α-naphthalene acetic acid, 0.1 mg/l 6-benzylaminopurine, 50 μg/ml hygromycin, 100 μg/ml carbenicillin and 250 μg/ml cefotaxime, and 0.8% phyto agar) in order to promote callus growth, select transformed plants and to prevent further growth of Agrobacterium. Elongated shoots were excised from calli and transferred on to 1/2 MS agar supplemented with 0.1 mg/L indole-3-acetic acid, 50 mg/L hygromycin and 100 mg/L carbenicillin in order to promote roots formation. Transformed plants were grown at 25°C in 16 h of light in axenic conditions without antibiotics and propagated every 5–6 weeks.

### Transient expression tobacco protoplasts and analysis of proteins

Protoplasts were isolated from tobacco leaves and subjected to polyethylene glycol-mediated transfection, as described by Pedrazzini et al. (1997). Forty micrograms of pDHA plasmid without insert (as a negative control) or with inserted recombinant coding sequences (pDHA-zein-hBMP2 and pDHA-hBMP2nat) were used in each transfection at a concentration of 10^6^ cells/mL. Protoplasts were then allowed to recover overnight in the dark at 25°C in K3 medium (Gamborg's B5 basal media with minimal organics supplemented with 400 mM sucrose, 1.5 M xylose, 3 mM NH4NO3, 1 mg/l α-naphtalenacetic acid, 1 mg/l 6-benzylaminopurine and 5 mM CaCl_2_) before performing protein extraction.

Protoplast homogenization was performed by adding to frozen samples two volumes of ice-cold homogenization buffer (150 mM Tris-Cl, 150 mM NaCl, 1.5 mM EDTA, and 1.5% Triton X-100, pH 7.5) supplemented with Complete® protease inhibitor cocktail (Roche, Basel). For protein extraction performed in reducing condition, the buffer was supplemented also with 4% 2-mercaptoethanol. Equal amounts of each sample were denatured with Laemmli buffer, loaded on to 15% SDS-PAGE together with the Protein Molecular Weight Marker mixture (Fermentas, Vilnius, Lithuania) and electrotransferred to a polyvinylidene difluoride (PVDF) membrane (Protran Nitrocellulose Transfer Membranes, Perkin Elmer). Blots were probed with anti-FLAG mAbs and proteins were detected using a horseradish peroxidase-conjugated anti-rabbit secondary antibody (Pierce Biotechnology, Rockford, IL), followed by chemiluminescence with Super-Signal® West Pico Chemiluminescent Substrate (Pierce Biotechnology, Rockford, IL). Detection of bands was performed with the ChemiDoc MP Imaging Systems (Bio-Rad, Hercules, CA).

### Protein extraction from tobacco leaves and western blot analysis

Screening of tobacco transgenic lines expressing zein-hBMP2ad or hBMP2nat was performed by direct homogenization of leaves (0.1 mg, fresh weight) in Laemmli buffer at 95°C (ratio 7:1). Twenty-five microliters of leaf homogenate from each sample (corresponding to 12.5 μg of total leaf proteins) were loaded on SDS-PAGE, followed by protein blot analysis with anti-Flag antiserum.

For the extraction of total proteins, young (5–7 cm long) leaves of transgenic tobacco were homogenized in an ice-cold mortar with seven volumes of homogenization buffer (200 mM NaCl, 1 mM EDTA, 0.2% Triton X-100, 100 mM Tris-Cl, pH 7.8) supplemented with Complete® protease inhibitor cocktail (Roche, Basel). For protein extraction performed in reducing condition, the buffer was supplemented also with 4% 2-mercaptoethanol. The homogenate was then centrifuged at 1500 g for 10 min at 4°C and the protein concentration of the supernatants was determined using the Bio-Rad protein assay (Bio-Rad, Hercules, CA, USA). Supernatant and, in some cases, pellet were then analyzed by protein blot. Equal amounts of total protein from each sample were denatured with Laemmli buffer and then processed as described above for protoplasts protein extracts. Densitometric analysis of bands was performed with Image Lab software (Version 4.1, Bio-rad).

### Endoglycosidase H treatment

Protein samples extracted either from transgenic leaves or from transfected protoplasts were incubated in Glycoprotein Denaturing Buffer (0.5% (w/v) SDS, 40 mM) for 10 min at 100°C. Samples were brought to a volume of at least 20 μl by adding of 10x G5 Reaction Buffer (50 mM sodium citrate pH 5.5) and divided in two equal aliquots. One of them was treated with 2000 Units of Endo H enzyme (New England Biolabs, Beverly, MA, USA), the other one with an equal volume of water. After 2 h incubation at 37°C the samples were supplemented with Laemmli denaturation buffer and analyzed by either SDS-PAGE and protein blotting.

### Velocity centrifugation on sucrose gradient

Young leaves of transgenic and control plants were homogenized using the above described homogenization buffer in absence of 4% 2-mercaptoethanol. The homogenate was loaded on a linear 5–25% (w/v) sucrose gradient made in 150 mM NaCl, 1 mM EDTA, 0.1% Triton X-100, 50 mM Tris-Cl, pH 7.5. An additional gradient was loaded with a mixture of protein markers containing 200 μg each of cytochrome C (12.4 kDa), ovalbumin (43 kDa), BSA (66 kDa), aldolase (161 kDa), and catalase (232 kDa). After centrifugation at 200,000 g for 20 h at 4°C in a Beckman SW40 rotor, fractions of about 650 μl were collected. An equal aliquot of each fraction was analyzed by SDS-PAGE and protein blot.

### Subcellular fractionation on isopycnic sucrose gradients

Young leaves of transgenic and wild type plants were homogenized in an ice-cold mortar with a 7:1 (v/w) ratio of homogenization buffer (100 mM Tris-Cl pH 7.8, 10 mM KCl, 12% sucrose (w/w), and Complete® protease inhibitor cocktail) containing either 1 mM EDTA or 10 mM MgCl_2_. Six hundred microliters of each sample were loaded on the top of 12 mL of liner sucrose gradient [100 mM Tris-Cl pH 7.8, 10 mM KCl, 16–65% sucrose (w/w)] and centrifuged at 150,000 g for 2 h at 4°C in a Beckman SW40 rotor (Beckman, Fullerton, CA, USA). After centrifugation, 20 fractions of about 600 μL were collected and an equal amount of each fraction was denatured and analyzed by SDS-PAGE and protein blot.

### Thrombin cleavage

Twenty-five micrograms of leaf total proteins, extracted in reducing condition from transgenic and wild type plants, were incubated with 10 units of thrombin (Amersham Biosciences, Piscataway, NJ, USA) or PBS (as control) at 22°C for 20 h, under gentle agitation. After digestion, samples were denatured and analyzed by SDS-PAGE and protein blot.

## Results

The fusion construct zein-hBMP2ad (Figure 1) contains the first 112 amino acids of 27 kD γ-zein, including its 19aa signal peptide, (Mainieri et al., 2004; de Virgilio et al., 2008; Virgili-López et al., 2013) followed by the C-terminal, active domain of hBMP2 (ad, 114 amino acids). The zein portion includes six out of the seven unpaired cysteine residues as well as the VHLPPP amphipathic heptapeptide. This is the same domain used in Mainieri et al. (2004) and it leads to the assembly of zeolin into PBs. A short flexible linker and a thrombin cleavage site were inserted between the fusion partners, to favor the independent folding of the two moieties and hBMP2ad purification (Figure 1). The full length native hBMP2 (hBMP2nat) pre-pro-sequence (which includes the hBMP2 signal peptide and the following pre-pro peptide) was also expressed, as a control. A C-terminal FLAG epitope was added to both recombinant constructs, to allow immunodetection (Figure 1).

### zein-hBMP2ad and hBMP2nat are soluble and are not secreted in tobacco mesophyll protoplasts

**Figure 2:**
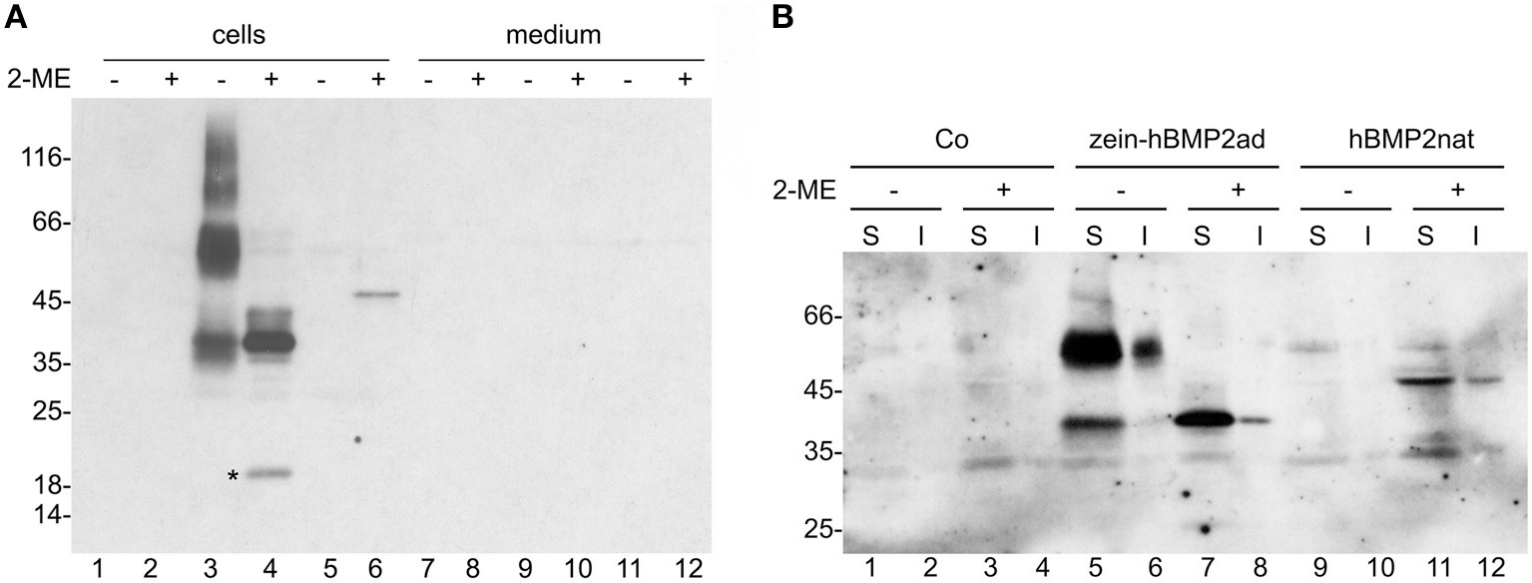
**hBMP2nat and zein-hBMP2ad are soluble in transiently transfected protoplasts and are not secreted**. **(A,B)** Tobacco leaf protoplasts were transiently transfected with plasmid encoding hBMP2nat (lanes 5–6, 11–12), zein-hBMP2ad (lanes 3–4, 9–10), or empty plasmid lanes (1–2, 7–8) and incubated for 24 h. **(A)** Protoplasts or incubation media were homogenized in the presence (+) or absence (−) of 2-ME. Aliquots corresponding to 20.000 protoplasts (cells) or the corresponding incubation medium (medium) were analyzed by SDS-PAGE in reducing conditions, followed by protein blot with anti-Flag antiserum. **(B)** Aliquots corresponding to 16.500 protoplasts were homogenized in the presence (+) or absence (−) of 2-ME. After centrifugation, soluble (S) material and insoluble precipitate (I) were analyzed by SDS-PAGE in reducing conditions, followed by protein blot with anti-Flag antiserum. Asterisk: 18 kD fragment of hBMP2ad. Numbers on the left indicate the positions of molecular mass markers, in kD.

The two constructs were transiently expressed in tobacco mesophyll protoplasts under the control of an enhanced cauliflower mosaic virus (CaMV) 35S promoter. The pDHA empty vector was used as further control (Co). The influence of disulphide bonds on solubility can be tested by protoplasts homogenization with buffer containing or not the reducing agent 2-mercaptoethanol (2-ME), followed by centrifugation to separate soluble and insoluble proteins. Solubilized proteins were then denatured in the presence of SDS and 2-ME and analyzed by SDS-PAGE in reducing conditions followed by protein blot with anti-Flag antibodies. When homogenization was performed in the absence of 2-ME, zein-hBMP2ad is recovered mainly as soluble form of about 55 kD that could represent dimers particularly difficult to denature (Figure 2A, lane 3). Higher molecular mass forms were also detected, as well as a polypeptide of about 37 kD. When homogenization was performed in the presence of 2-ME, the 37 kD form was by far the major one detected, indicating that it represents the monomeric form, which remains strongly assembled into dimers and further polymers when the reducing agent is not present at the time of homogenization (Figure 2A, lane 4). An 18 kD polypeptide was also detected, probably derived by proteolytic cleavage occurring in the C-terminal region.

hBMP2nat migrated as a polypeptide of 45 kD, as expected, but it accumulated at much lower levels than the zein fusion and was soluble only in the presence of 2-ME (Figure 2A, lanes 5, 6). Neither zein-hBMP2ad nor hBMP2nat were detectable in the protoplast incubation medium (Figure 2A, lanes 7–12), strongly suggesting that the two proteins were not secreted.

To verify whether a proportion of protein remains insoluble even when homogenized in the presence of 2-ME, after homogenization in oxidizing or reducing conditions samples where centrifuged and both soluble (S) and insoluble (I) fractions were denatured and analyzed by SDS-PAGE and protein blot (Figure 2B). Only a small amount of zein-hBMP2ad was insoluble, both in the presence and absence of 2-ME (Figure 2B, lanes 6, 8), confirming that this recombinant protein is almost completely soluble in aqueous buffer. A higher proportion of hBMP2nat (about 20%) remained insoluble after homogenization with the reducing agent (Figure 2B, compare lanes 11 and 12). When homogenization was performed without 2-ME, hBMP2nat was not recovered, neither in the soluble fraction nor as an insoluble precipitate (Figure 2B, lanes 9–10), indicating that tissue homogenization without the reducing agent could lead to the precipitation of insoluble aggregates of hBMP2nat that cannot be further denatured.

### In transgenic plants, zein-HBMP2ad is retained in the ER mainly as soluble dimers and accumulates at higher levels compared to hBMP2nat

**Figure 3:**
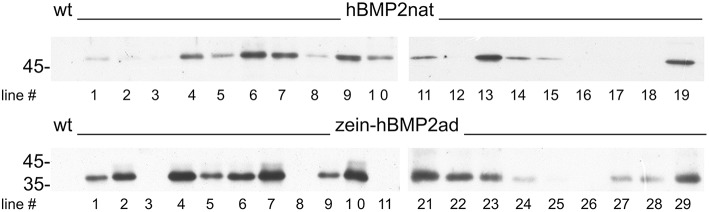
**zein-hBMP2ad accumulates to higher levels than hBMP2nat in transgenic plants**. Leaves (0.5 g, fresh weight) from independent transgenic tobacco plants expressing hBMP2nat (upper panel, lines 1–19), zein-hBMP2ad (lower panel, lines 1–11, 21–29) or untransformed plants (wt), were homogenized in Laemmli buffer at 95°C (ratio 7:1). Twenty-five microliters of leaf homogenate from each sample (corresponding to 12.5 μg of total leaf proteins) were subjected to SDS-PAGE, followed by protein blot analysis with anti-Flag antiserum. Protein blot exposure time: hBMP2nat, 15 min; zein-hBMP2, 1 min. Numbers on the left indicate the position and size (in kDa) of molecular mass markers.

Since both zein-hBMP2ad and hBMP2nat accumulated during transient expression, albeit at different levels, we produced tobacco transgenic plants expressing the two constructs under the CaMV35S promoter. Leaf extract from several hygromycin resistant putative transgenic lines grown in axenic conditions for 4–6 weeks were analyzed by protein blot with anti-Flag antiserum. Accumulation of the recombinant proteins was variable, as it nearly always happens when different transgenic plants are analyzed; however, zein-hBMP2ad showed a clear tendency to accumulate at much higher levels than hBMP2nat (Figure 3, compare upper and bottom panels, and notice that the hBMP2nat protein blot was exposed for a 15 times longer time than the zein-hBMP2ad one).

**Figure 4:**
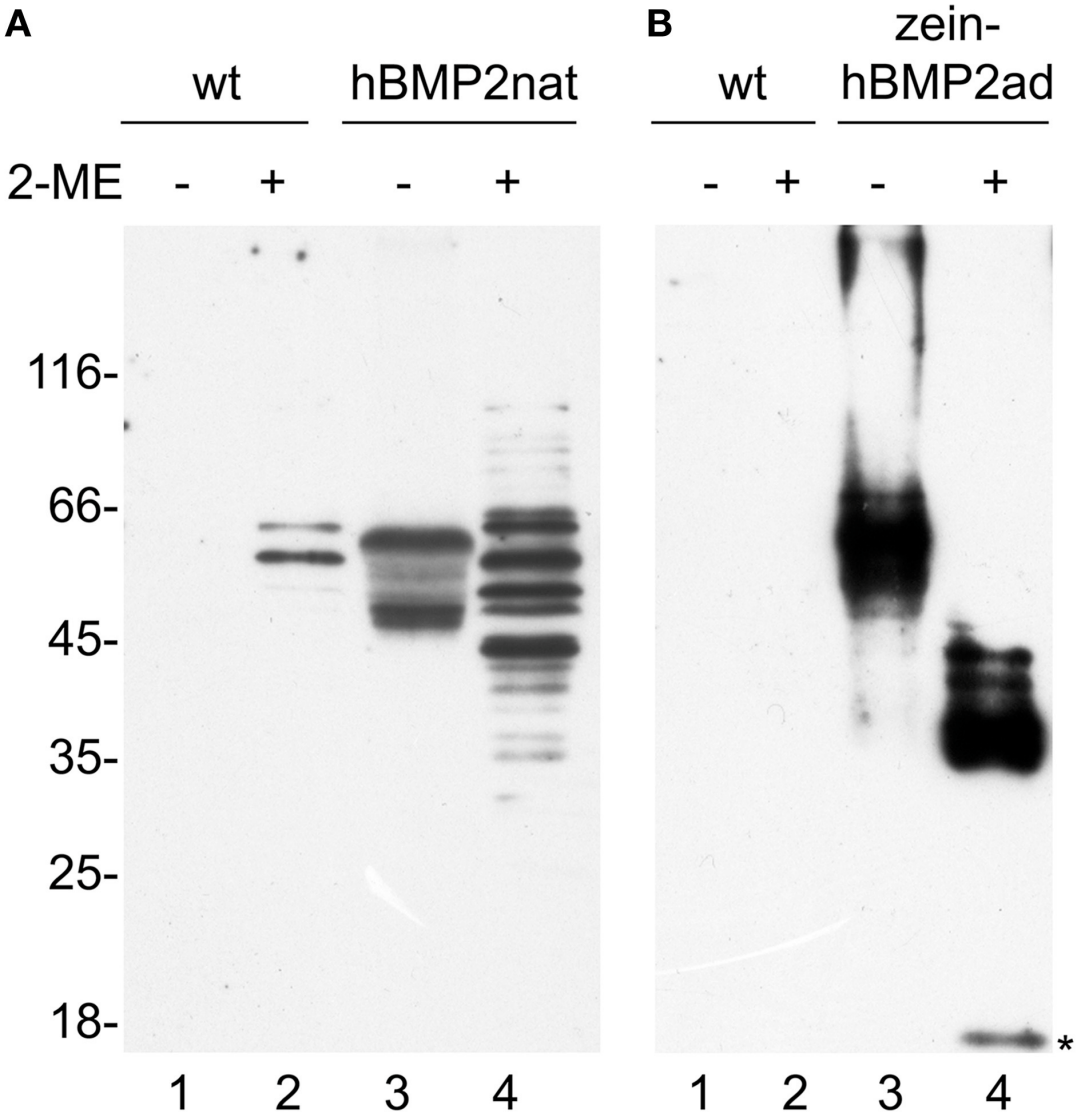
**Both hBMP2nat and zein-hBMP2ad are soluble in tobacco transgenic plants**. Thirty micrograms of total leaf proteins from wild type (wt, **A**, lanes 1–2) and hBMP2nat line #3 (**A**, lanes 3–4) plants or 3 μg of total leaf proteins from wild type (wt, **B**, lanes 1–2) and zein-hBMP2ad line #7 (**B**, lanes 3–4) plants, were extracted in the absence (−) or presence (+) of 2-ME and analyzed by SDS-PAGE and protein blot with anti-Flag antiserum. Asterisk: 18 kD hBMP2ad fragment. Numbers on the left indicate the positions of molecular mass markers, in kD.

The solubility grade of hBMP2nat and zein-hBMP2ad in transgenic plants was tested by homogenization in either oxidizing or reducing conditions. The banding pattern as well as the solubility of zein-hBMP2ad were very similar to those obtained upon transient expression (Figure 4B, lanes 3, 4, and compare with lanes 3 and 4 in Figure 2A), with most of the protein soluble in the absence of 2-ME. When hBMP2nat leaf homogenization was performed in the absence of reducing agent, a polypeptide of the expected 45 kD molecular mass was detected (Figure 4A, lane 3), as well as a larger form around 55 kD that could indicate heterogeneous N-glycosylation (Hang et al., 2014, and see below). However, in the presence of 2-ME, instead of these two main polypeptides several other forms were detected (Figure 4A, lane 4), that became also visible in transient expression by longer exposure of the blot in Figure 2A (see Figure S1, lanes 5, 6), suggesting the presence of intrachain disulfide bonds and/or partial proteolysis. Two of these polypeptides were also observed in extracts from untransformed plants (Figure 4A, lane 2), and represented endogenous tobacco proteins, visible because of the relatively high amount of total protein loaded to clearly visualize the hBMP2nat polypeptides. Notice that in Figure 4 one tenth of the total amount of leaf protein was analyzed in Figure 4B compared to Figure 4A, because of the higher amount of zein-hBMP2ad accumulation with respect to hBMP2nat; for this reason the endogenous tobacco polypeptides recognized by the anti-Flag antiserum were not visible in Figure 4B.

We reasoned that the zein fusion could be more promising for the production of recombinant hBMP2ad because of its higher accumulation levels compared with those of the native osteogenic factor. We therefore focussed on zein-hBMP2ad for subsequent experiments.

**Figure 5:**
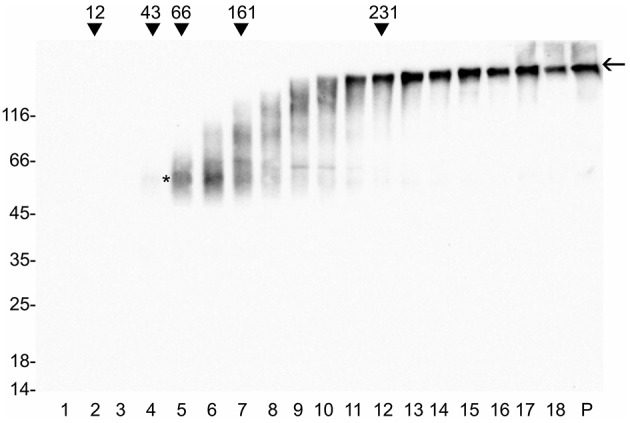
**zein-hBMP2ad assembles both in dimers and in progressively larger multimers**. Eight hundred micrograms of total leaf proteins from zein-hBMP2ad line #7 were extracted in the absence of 2-ME and fractionated by velocity sucrose (5–25% w/v) gradient centrifugation. Equal volume of fractions and the pellet at the bottom of the tube were analyzed by protein blot with anti-Flag antiserum. Asterisk indicates zein-hBMP2ad dimers. Arrow indicates the interface between stacking and separating gels. P: pellet at the bottom of the tube. Numbers on the top indicate the position along the gradient and the molecular mass (kD) of velocity centrifugation markers. Numbers on the left indicate the positions of molecular mass markers, in kD.

Previously published data showed that, in transgenic plants, full-length γ-zein or protein fusions containing its N-terminal domain were insoluble in the absence of reducing agent and were formed by very large disulfide-bonded polymers that migrated at the bottom of the tube under velocity centrifugation analysis (Bellucci et al., 2000; Mainieri et al., 2004; Torrent et al., 2009a; Virgili-López et al., 2013). Conversely, our results showed that zein-hBMP2ad was similarly soluble in oxidizing and reducing conditions. We therefore investigated its polymerization grade. Total leaf homogenate from zein-hBMP2ad line #7 (see Figure 3, bottom panel) was extracted in the absence of 2-ME and fractionated by velocity sucrose (5–25% w/v) gradient ultracentrifugation. Equal proportions of each fraction and of the pellet at the bottom of the tube (solubilized by SDS-PAGE denaturation buffer) were analyzed by protein blot with anti-Flag antiserum (Figure 5). zein-hBMP2ad migrated both as dimers (Figure 5, asterisk) and as multimers that fractionated at progressively distant positions along the gradient until the bottom of the tube, indicating the formation of very large polymers that were nevertheless still soluble. Only a very small proportion ended up at the bottom of the tube; this pellet could contain insoluble polymers. As the size of the polymers increased and exceeded the 231 kD marker, these could no longer be disassembled by the SDS-PAGE and migrated progressively more slowly. The larger ones actually remained at the interface between the stacking and separating gels (Figure 5, arrow).

**Figure 6:**
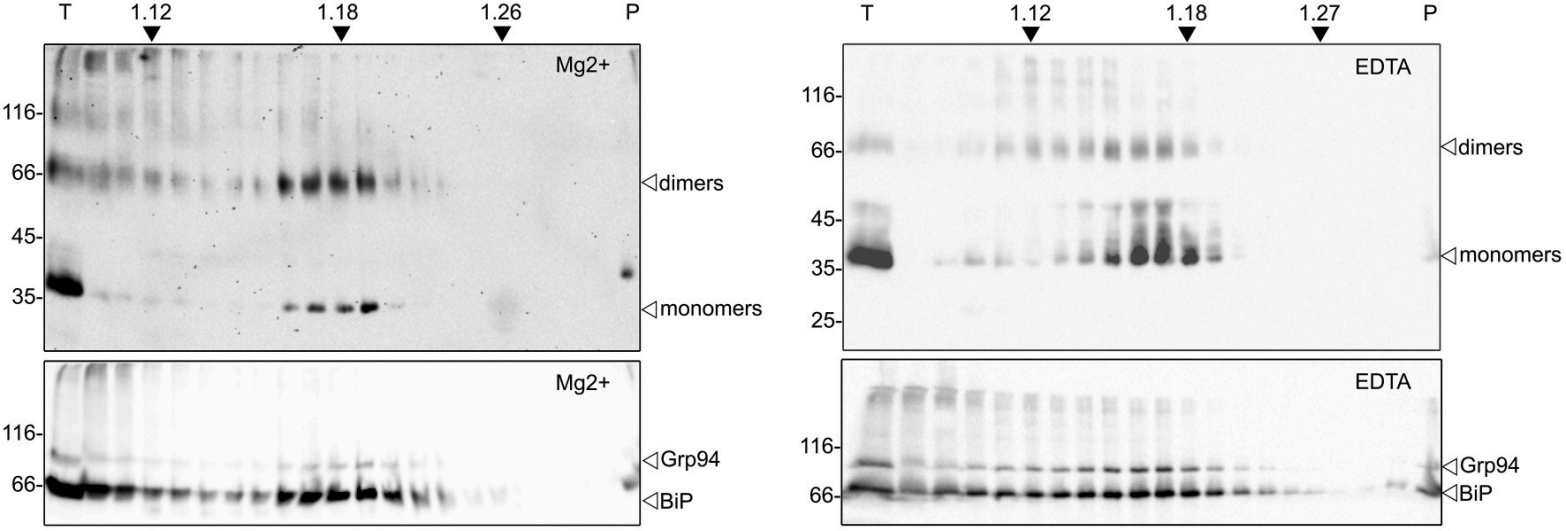
**zein-hBMP2ad cofractionates with the ER**. Hundred milligrams of fresh leaves from zein-hBMP2ad line #23 were homogenized in sucrose buffer, in the absence of detergent. The homogenates were fractionated by centrifugation on isopycnic sucrose gradient (16–65% w/w) in the presence of MgCl_2_ (left panels) or EDTA (right panels). Collected fractions were analyzed by SDS-PAGE followed by immunoblotting with anti-Flag antiserum (upper panels) or with a mix of anti-BiP and anti-Grp94 antisera (bottom panels). T, total extract; P, pellet at the bottom of the tube. Numbers at top indicate the density (g/ml) of sucrose. Numbers at left indicate the position of molecular mass markers, in kD.

The intracellular localization of zein-hBMP2ad was investigated by subcellular fractionation on isopycnic sucrose density gradient. Because the binding of ribosomes to the ER membrane is dependent on Mg2+, the chelation of this cation with ethylenediaminetetraacetic acid (EDTA) disengages the ribosomes from the membrane, determining a density shift of the ER-derived microsomes, and not of other compartments, to lighter fractions of the gradient. Leaves from zein-hBMP2ad (line #23) were homogenized in the absence of detergent and in the presence of sucrose to maintain organelle integrity. The homogenate was loaded on two 16–65% (W/W) sucrose gradients containing Mg2+ or EDTA and subjected to centrifugation. Equal amount of each fraction were analyzed by SDS-PAGE followed by protein blot with either anti-Flag serum or sera against the ER markers BiP and endoplasmin (Grp94). In the presence of Mg2+, zein-hBMP2ad was mainly detected in four fractions with a peak around a density of 1.18–1.19 mg/mL, where the ER marker BiP and Grp94 also peaked (Figure 6, panels on the left). The presence of EDTA caused a shift of both zein-hBMP2ad, BiP and Grp94 to lower density fractions (peaks around 1.16–1.17 mg/mL), coherent with the density shift of the ER due to ribosome release (Figure 6, panels on the right). The ER marker BiP and, in minor proportion Grp94 and zein-hBMP2ad, were also present at the top of the gradient, as observed previously in similar experiments (Pedrazzini et al., 1997; Mainieri et al., 2004), possibly reflecting partial release from the ER lumen on homogenization.

Taken together, our results indicate that the 27 kD γ-zein N-terminal domain is sufficient to retain zein-hBMP2ad in the ER, but it does not allow the hyper-polymerization and insolubility that are typical of protein bodies formed by zein in maize endosperm and zeolin in transgenic tobacco.

### zein-hBMP2ad is glycosylated in tobacco transgenic plants

Native hBMP2 contains five putative N-glycosylation sequons: four of them occur within the protein prosegment domain (positions N135, N163, N164, N200), and one in the active domain (position N338). It has been recently demonstrated that in both CHO and HEK293T cells, the positions N135, N200, and N338 are actually glycosylated (see Figure 1), suggesting that the N-glycosylation status of hBMP2 is independent of cell types and species (Hang et al., 2014). Moreover, the three N-glycans are sensitive to peptide-N-glycanase F (PNGase F, which removes both high-mannose and Golgi-modified, complex type N-glycans) and Endoglycosidase H (Endo H, which removes only high-mannose glycans), indicating that they are of the high-mannose type. The N-terminal region of 27 kD γ-zein does not contain N-glycosylation sites.

**Figure 7:**
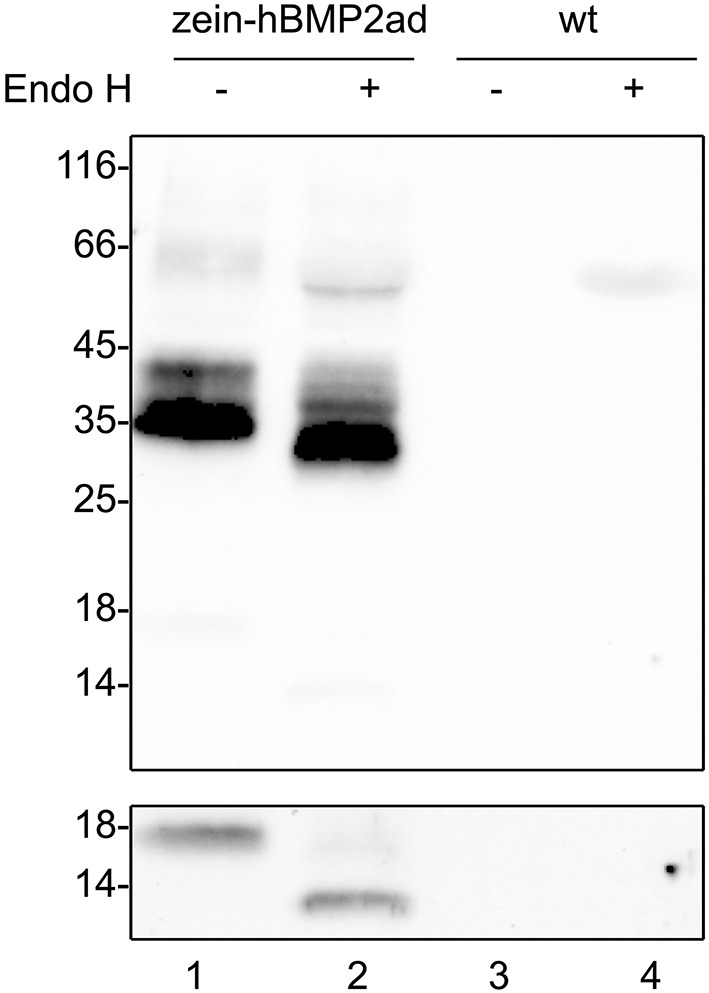
**zein-hBMP2ad is N-glycosylated**. Eight micrograms of total leaf proteins from zein-hBMP2ad line #7 or from wt plants, extracted in the presence of 2-ME, were incubated with Endo H (+) or without enzyme (−) as control and analyzed by SDS-PAGE and protein blot with anti-Flag antiserum. Bottom panel: longer exposure of the 14–18 kD portion of the blot. Numbers at left indicate the position of molecular mass markers, in kD.

To asses whether the N-glycosylation at position N338 of the active domain was maintained in zein-hBMP2ad transgenic plants, total leaf proteins form line #7 were extracted in the presence of 2-ME and incubated with or without Endo H. Treatment with the endoglycosidase caused an electrophoresis mobility shift, consistent with the removal of one high-mannose N-linked glycan (Figure 7, compare lane 1 and 2). Upon long exposure of the blot, the 18 kD fragment was detectable and was also sensitive to Endo H digestion (Figure 7, bottom panel, compare lane 1 and 2). Taken together, the results indicate that in transgenic plants the N338 position of hBMP2ad is N-glycosylated and the 18 kD fragment is most probably the active domain that has been cleaved from the γ-zein moiety.

### The hBMP2 active domain can be efficiently recovered by thrombin cleavage

It has been previously observed that the accumulation levels of recombinant proteins in transgenic plants could depend on leaf age and size, with younger leaves showing better performance than the older ones (McCabe et al., 2008; Kolotilin et al., 2013). The size and position of leaves along the stem identify their age: older and larger leaves are positioned below newer ones. To analyze zein-hBMP2ad accumulation in relationship to leaf age, total proteins were extracted without 2-ME from leaves (line #7, see Figure 3) with different size (from the bottom to the apex). Younger leaves accumulate higher amounts of zein-hBMP2ad per mg of total protein (Figure S2, lanes 2, 9). In the light of these results, 3–4 cm long leaves from young plants were used to quantify the hBMP2ad produced by tobacco plants.

**Figure 8:**
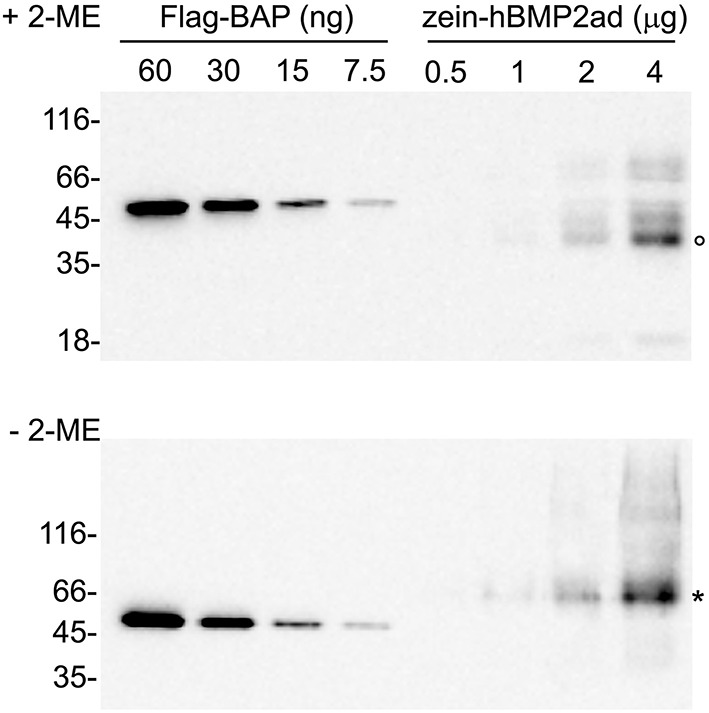
**Quantification of zein-hBMP2ad expressed in tobacco plants**. Sequential dilutions of total leaf proteins (from 0.5 to 4 μg) from zein-hBMP2ad line #23 extracted in the presence (+2-ME) or in the absence (−2-ME) of 2-mercaptoethanol were analyzed by protein blot in comparison with known amounts of the commercial standard protein Flag–BAP (lanes on the left). Empty circle: zein-hBMP2ad monomers; asterisk: zein-hBMP2ad dimers. Numbers at left indicate the position of molecular mass markers, in kD.

A preliminary quantification of the full-length zein-hBMP2ad was performed by densitometric analysis of the bands by comparing progressive dilutions of total leaf proteins from line #23 (see Figure 3), extracted in the presence or absence of 2-ME (Figure 8, upper and lower panels, respectively) with known amounts of the commercial standard protein Flag–BAP, (Figure 8, lanes on the left). The results indicated that full-length zein-hBMP2ad could represents about 1.1 and 1.25% of total soluble proteins extracted with or without 2-ME, respectively. Densitometric quantification of zein-hBMP2ad full-length from other independent lines showed that the accumulation levels reached the 1.75 and 2.25% in plants #7 and #27, respectively (Figure S3), indicating a certain grade of variability (see Figure S2).

**Figure 9:**
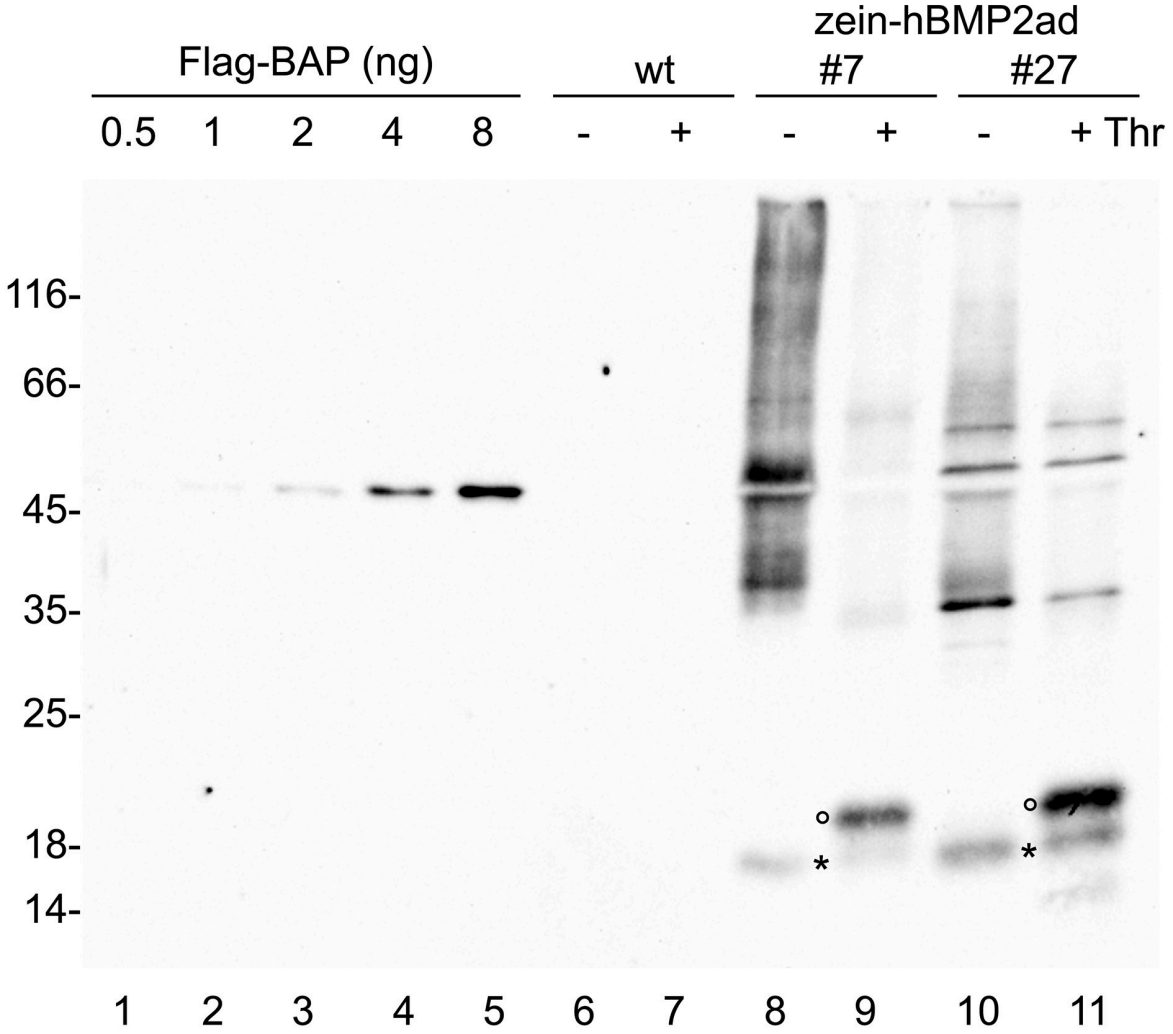
**The hBMP2 active domain is efficiently released from zein-hBMP2ad by thrombin cleavage**. Twenty-five micrograms of total leaf proteins, extracted in the presence of 2-ME from zein-hBMP2ad transgenic (lines #7 and 27) or wt plants, were incubated for 20 h in the presence (+) or absence (−) of thrombin. The reaction mixtures were analyzed by SDS-PAGE, followed by protein blot using anti-Flag antiserum. hBMP2ad was quantified by comparison with known amounts of the commercial standard protein Flag–BAP (lanes on the left). Empty circles: 20 kD hBMP2ad. Asterisks: 18 kD fragment of hBMP2ad. Numbers at left indicate the position of the molecular weight markers, in kDa.

To investigate the accessibility of the thrombin cleavage site to the protease and to quantify the yield of hBMP2ad, total leaf proteins were extracted in reducing conditions from wild-type or two different zein-hBMP2ad plants (#7 and #27) and subjected to digestion with thrombin or incubated in the absence of the enzyme for 20 h. Samples were analyzed by SDS-PAGE and protein blot with anti-Flag antiserum (Figure 9, lanes 6–11). Under mock treatment, zein-hBMP2ad monomers, dimers and polymers are observed (Figure 9, lanes 8, 10); the 18 kD proteolytic fragment was also present (Figure 9, lanes 8, 10, asterisks). The fusion protein zein-hBMP2ad was efficiently cleaved by thrombin and the hBMP2ad portion was mostly released as a 20 kD fragment (Figure 9, lanes 9, 11, empty circles), corresponding to the expected molecular mass of the N-glycosylated active domain. The exact efficiency of cleavage is not easy to establish, because of the smeared electrophoretic pattern of the undigested protein, but clearly the vast majority of polypeptides have been successfully cleaved. The 18 kD fragment did not change electrophoretic mobility upon thrombin treatment, indicating that its N-terminus is downstream the thrombin cleavage site and upstream the N-glycosylation site (Figure 9, asterisks, compare lanes 8–9 and 10–11). Quantification of the unpurified hBMP2ad fragment performed by comparison with known amounts of the commercial standard protein Flag–BAP (Figure 9, lanes 1–5) indicated that hBMP2ad represents around 0.02% of total soluble proteins extracted in the presence of 2-ME.

A more precise quantification of intact zein-hBMP2ad or released hBMP2ad will be necessary to identify the best expressor among the different lines. The quantification should be performed by ELISA technique using anti-hBMP2 antibodies.

## Discussion

### zein-hBMP2ad provides information on the requirements for PB formation

Several studies have reported that the γ-zein N-terminal domain, can successfully lead to high accumulation via PB formation when fused to the N- or C-terminus of other proteins introduced into the ER, such as bean phaseolin (Mainieri et al., 2004), HIV-p24 (Virgili-López et al., 2013), xylanase (Llop-Tous et al., 2011), Moreover, this system can be used not only in plant tissues or cell cultures but also in fungal, insect, and mammalian cells (Torrent et al., 2009b). Previous results showed that zeolin, the chimeric protein obtained by attaching the γ-zein N-terminal domain at the C-terminus of phaseolin, which is a vacuolar seed storage protein, has all major features of zein: ER retention, PB formation and insolubility in absence of reducing agents (Mainieri et al., 2004). When this γ-zein domain was fused to the cytosolic viral protein Nef, it was unable to prevent degradation by the ER quality control (de Virgilio et al., 2008). However, Nef was stabilized and formed PBs when the entire zeolin sequence was fused to it. Altogether, these data suggest that specific structural characteristics of the non-zein portion may influence the fate of chimeric proteins containing the γ-zein N-terminal domain. Phaseolin, which like other storage proteins has a tendency to form large complexes, may contribute to promote protein packaging accelerating the process of PBs formation. Conversely, Nef is a cytosolic protein; when introduced into the oxidizing ER environment its cys residues could negatively affect correct polymerization by the zein domain, leading to rapid entry into the degradative pathway of ER quality control before PB formation could occur. The results presented here suggest that the seven cysteine residues of hBMP2ad do not have a destabilizing effect on zein-hBMP2ad and actually the chimera accumulates to higher amounts than native hBMP2, which is a protein naturally introduced into the ER. However zein-hBMP2ad remains soluble also in the absence of reducing agents and in large proportion it does not undergo the extensive, polymerization and insolubilization events that are typical of protein bodies formed by zein in natural maize seeds and by zeolin in transgenic plants: velocity gradient centrifugation showed that most of the chimera forms soluble dimers and oligomers below 231 kD. This is perhaps an unexpected result, and constitutes a case in which the zein domain fails to promote insolubilization but, nevertheless, leads to retention and stabilization of a protein introduced into the ER. The C-terminal region of the real 27 kD γ-zein (the 2S albumin-like domain, Mainieri et al., 2014) contains six cysteines paired in three intra-chain disulphide bonds. hBMP2ad has one more cysteine that is engaged in the dimer formation. The position of this inter-chain disulphide bridge may disturb the close packing that leads to insolubility. It should also be noticed that we did not detect secretion of hBMP2nat upon transient expression. This unexpected result may indicate that anyway, at least in plant cells, this protein does not efficiently enter intracellular traffic along the secretory pathway and, in the absence of the zein stabilizing domain may be degraded by ER quality control.

Subcellular fractionation indicates that zein-hBMP2ad accumulates in the ER. Its single N-linked glycan can be removed by endoglycosidase H, indicating that it is not processed by Golgi-enzymes; itself, this is not a demonstration of lack of trafficking along the secretory pathway, because protein folding can inhibit access of Golgi glycosyltransferases, but it is certainly consistent with an ER localization. Indeed, it has been recently demonstrated that in CHO cells all the three hBMP2 N-glycans are EndoH sensitive, even if the protein traffics through the Golgi complex and is secreted (Hang et al., 2014). The N-glycan of hBMP2ad might also increase solubility of the chimeric protein, preventing protein body formation. Phaseolin present in zeolin also has one N-linked glycan, but this clearly does not impair the formation of insoluble protein bodies (Mainieri et al., 2004). If the glycan of hBMP2ad plays a role in preventing insolubility, one possible explanation for this discrepancy could reside in the fact that the phaseolin glycan is exposed on the protein surface, as demonstrated by its accessibility to Golgi enzymes (Sturm et al., 1987), whereas that of hBMP2ad is probably masked by interactions with the polypeptide chain, since it does not become Golgi-modified *in vivo*. Such different interactions between N-glycans and the polypeptide chains can exert different influence on the ability of the chimeric proteins to assemble into protein bodies.

The question therefore arises of how zein-hBMP2ad is retained in the ER, in spite of not having a H/KDEL signal for ER retention and failing to form insoluble large polymers. Pioneering work on the mechanism of 27 kD γ-zein retention in the ER showed that the wild-type protein was able to form PB in the ER of vegetative plant tissues (Shewry et al., 1995), but neither of the two domains, each corresponding approximately to half of the protein, could (Geli et al., 1994). The 27 kD γ-zein C-terminal domain, engineered to enter the ER by the addition of a signal peptide, was secreted, while the N-terminal domain, containing its own signal peptide and all the seven cysteine residues, was soluble also in the absence of reducing agent and accumulated in the ER as diffuse, slightly electron-dense material, very different from the well-defined, round shaped and highly electron dense PB formed by the entire 27 kD γ-zein polypeptide (Geli et al., 1994). The ER retention of the N-terminal domain was hypothesized to occur because of the repeated PPPVHL hexapeptide. It was later shown (Kogan et al., 2002) that a synthetic version of the eight PPPVHL repeats forms an amphypatic helix and interacts with liposomes *in vitro*. It is therefore possible that the addition of the hBPM2ad sequence to the zein domain has a sort of “neutral” effect: it does not alter folding and therefore it does not lead to ER quality control degradation but at the same time it does not allow extensive interactions among the Cys residues of the N-terminal zein domain that are necessary for the formation of the insoluble PB. Therefore, zein-BMP2ad would behave like the γ-zein N-terminal domain expressed alone, consistent with the hypothesis that the structural characteristics of the C-terminal domain of this prolamin also play a role in PB formation.

### Cost-benefit in using γ zein-fusion for the production of hBMP2ad in transgenic plants

Actually, rhBMP2 for clinical purpose were produced from Medtronic in CHO DHFR-deficient cell lines via methotrexate-mediated gene amplification (Israel et al., 1992). The consumption of time and the high costs of the purification steps, added to the low yield of the final product (ng/ml) make more difficult the widespread use of recombinant rhBMP2 in clinical applications (Lee et al., 2010; Luan et al., 2011). After the FDA approval, many attempts have been made to produce rhBMP2 in the less expensive bacterial systems, as an alternative to mammalian cells (Vallejo et al., 2002; Park et al., 2014). However, rhBMP2 is a secretory protein that needs the oxidizing environment of the eukaryotic ER lumen to fold and assemble correctly; moreover, rhBMP2 produced in *E. coli* does not undergo N-glycosylation, a protein modification that occurs cotranslationally in the ER lumen. Therefore, the biological activity of *E. coli*-derived rhBMP2 has to be restored by *in vitro* refolding after purification, adding some complexity to the production method (Kübler et al., 1998; Bessho et al., 2000). Despite a 110 mg/L production rate of *E. coli*-derived rhBMP2, its activity and efficacy after refolding is controversial (Bessho et al., 2000; Bessa et al., 2008; Yano et al., 2009; Lee et al., 2010). Recently, same preclinical studies showed promising results in using *E. coli*-produced rhBMP2 for the regeneration of experimentally induced bone defects (Harada et al., 2012; Ono et al., 2013; Chung et al., 2015; You et al., 2016), even if the most effective dose as well as the optimal carrier are still to be determined. A comparison between CHO- and *E. coli*-derived rhBMP2 using a dog critical-size supraalveolar peri-implant model showed that the two rhBMP2s are equally effective in inducing bone formation (Lee et al., 2013); however, no economic analysis was provided in this study. Up to now, a conclusive study on the costs-benefits, based on the comparison between the different methods of production of BMP-2 actually on the market, is not yet available.

In a previous study, Suo et al. (2006) reported the production of human BMP-2 active domain at level 0.02% TSP in tobacco plants. It is not easy to make a comparison of this production efficiency and our results, because the constructs used for tobacco transformation are different. In Suo et al. (2006), the maximum yield of hBMP2ad was obtained from plants transformed with a constructs containing a combination of double 35SCaMV promoter, AMV enhancer, and two Rb7 MARs sequences that promotes transgene expression. Plants transformed with the construct containing a single 35SCaMV promoter, without the MARS sequences, had about five times lower expression level (measured by GUS activity). Using a single 35SCaMV promoter, we reached the yield of about 0.02% TSP, which could be further improved by doubling the promoter or by addition of enhancer sequences. It should also be underlined that Suo et al. expressed hBMP2ad without the signal peptide; the osteogenic factor is therefore most likely accumulated in the cytosol, a non-optimal environment for a secretory protein.

A recent randomized clinical trial using *E. coli*-derived rhBMP2, reported the efficacy of 0.5–2 mg of 1 mg/ml rhBMP-2 in bone formation after maxillary sinus augmentation (Kim et al., 2015). Obviously, the total dose depends on the defect size (Boyne et al., 2005; Fiorellini et al., 2005; Herford and Boyne, 2008; Triplett et al., 2009). Once the initial cost of the F0 transgenic production has been overcome, plant-based systems do not require the same expensive investments as other production methods. From our best producing plant we recovered about six micrograms of hBMP2ad/g FW: a rough estimate suggests that one transgenic plant may be sufficient for a single clinical application. Only a comparative study between hBMP2 produced in plant, mammalian, or bacterial systems, will allow to make more precise estimates about cost-effectiveness, and to establish whether transgenic plants could really represent a valuable alternative to the currently availableproducts.

## Author contributions

EP supervised the entire work, designed all experiments and wrote the paper. DM contributed in designing the experiments. VC performed the experiments and contributed in writing the paper. MD contributed in writing the paper. RW obtained grant support.

### Conflict of interest statement

The authors declare that the research was conducted in the absence of any commercial or financial relationships that could be construed as a potential conflict of interest.
