# Supplementary figures and images for: A Fusion between Domains of the Human Bone Morphogenetic Protein-2 and Maize 27 kD γ-Zein Accumulates to High Levels in the Endoplasmic Reticulum without Forming Protein Bodies in Transgenic Tobacco
Source: Front Plant Sci. 2016 Mar 24;7:358. doi: 10.3389/fpls.2016.00358 (PMC4805588; doi:10.3389/fpls.2016.00358)

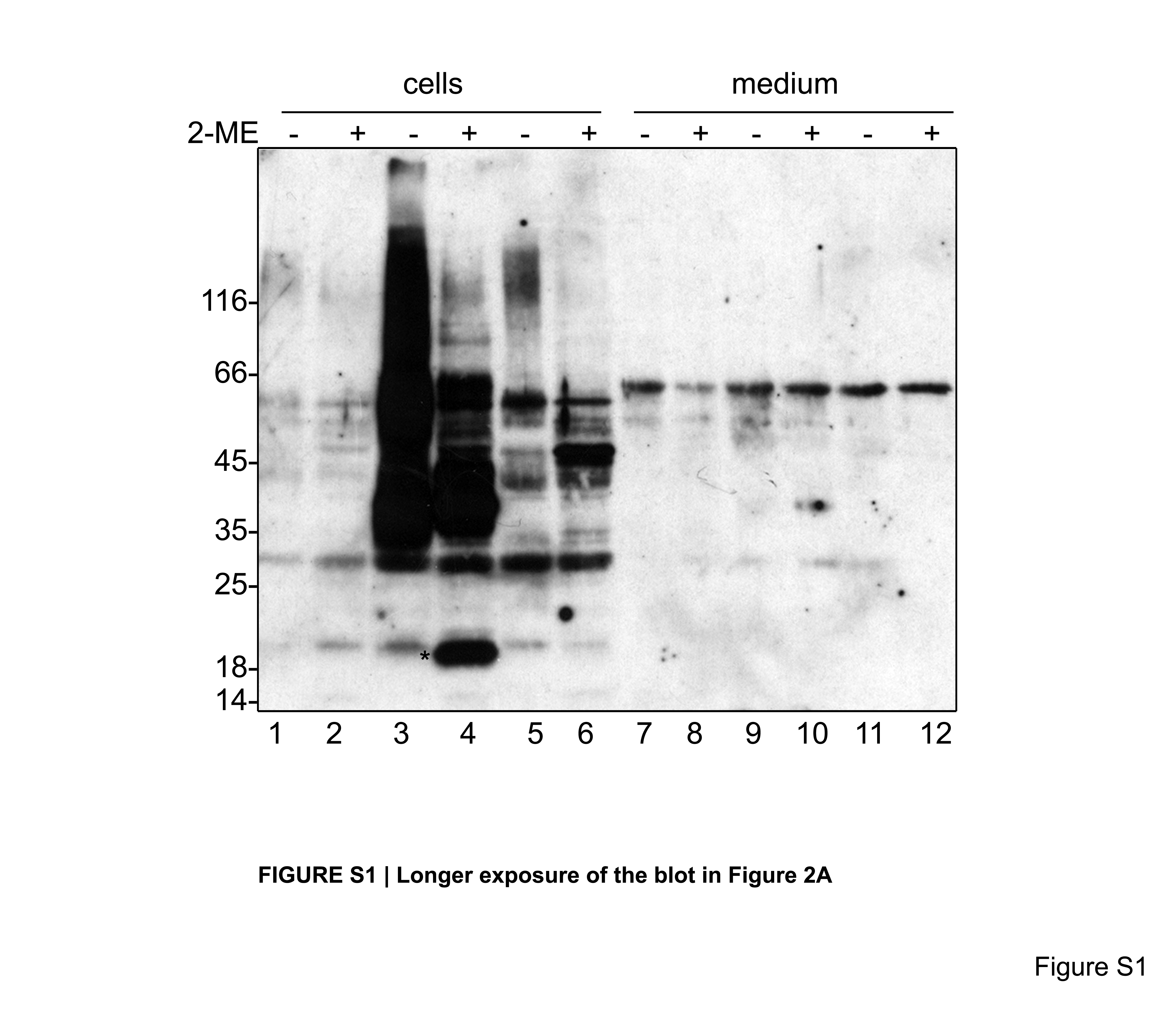

Supplement: Supplementary file 1 [file Image1.JPEG]

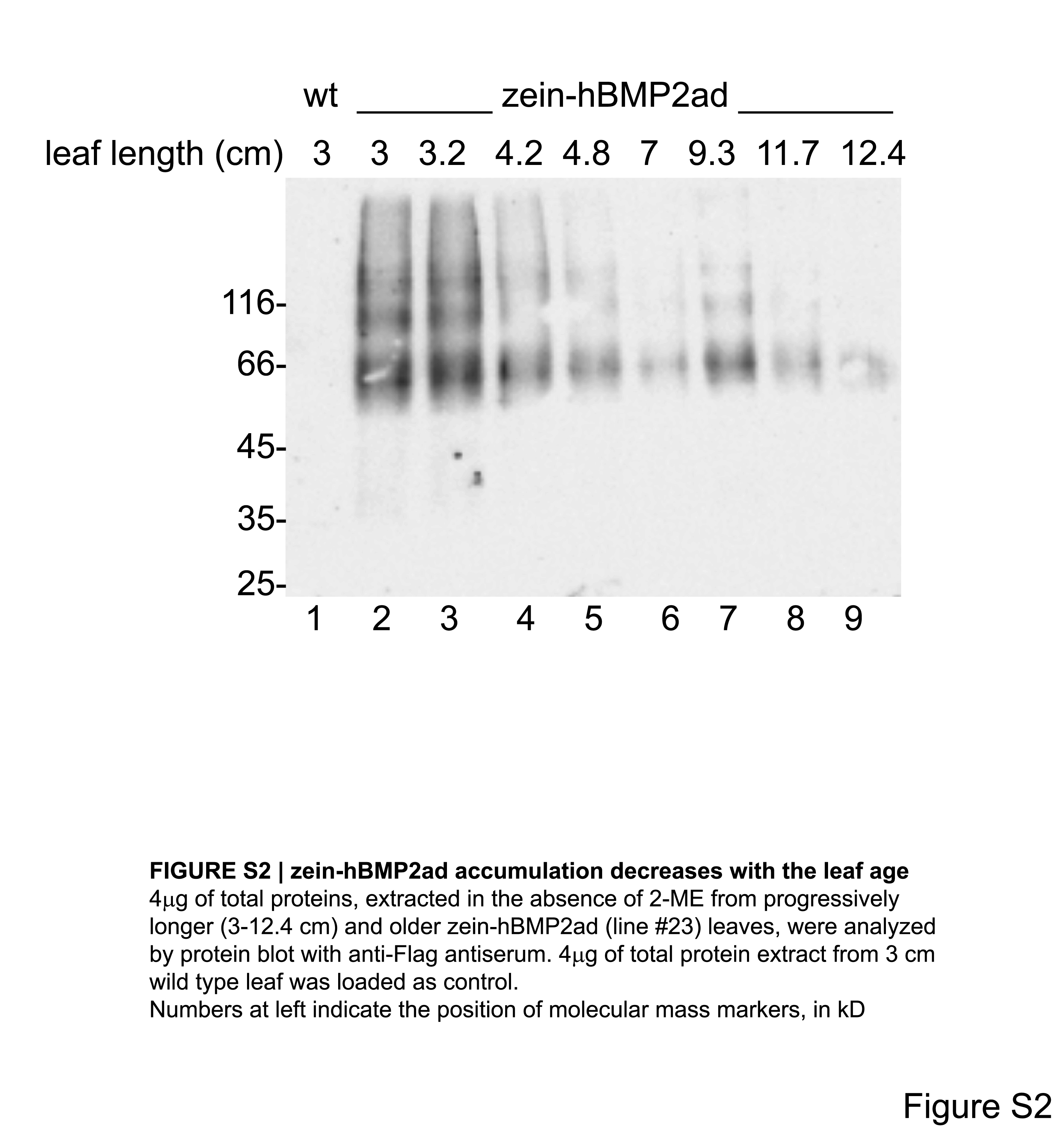

Supplement: Supplementary file 2 [file Image2.JPEG]

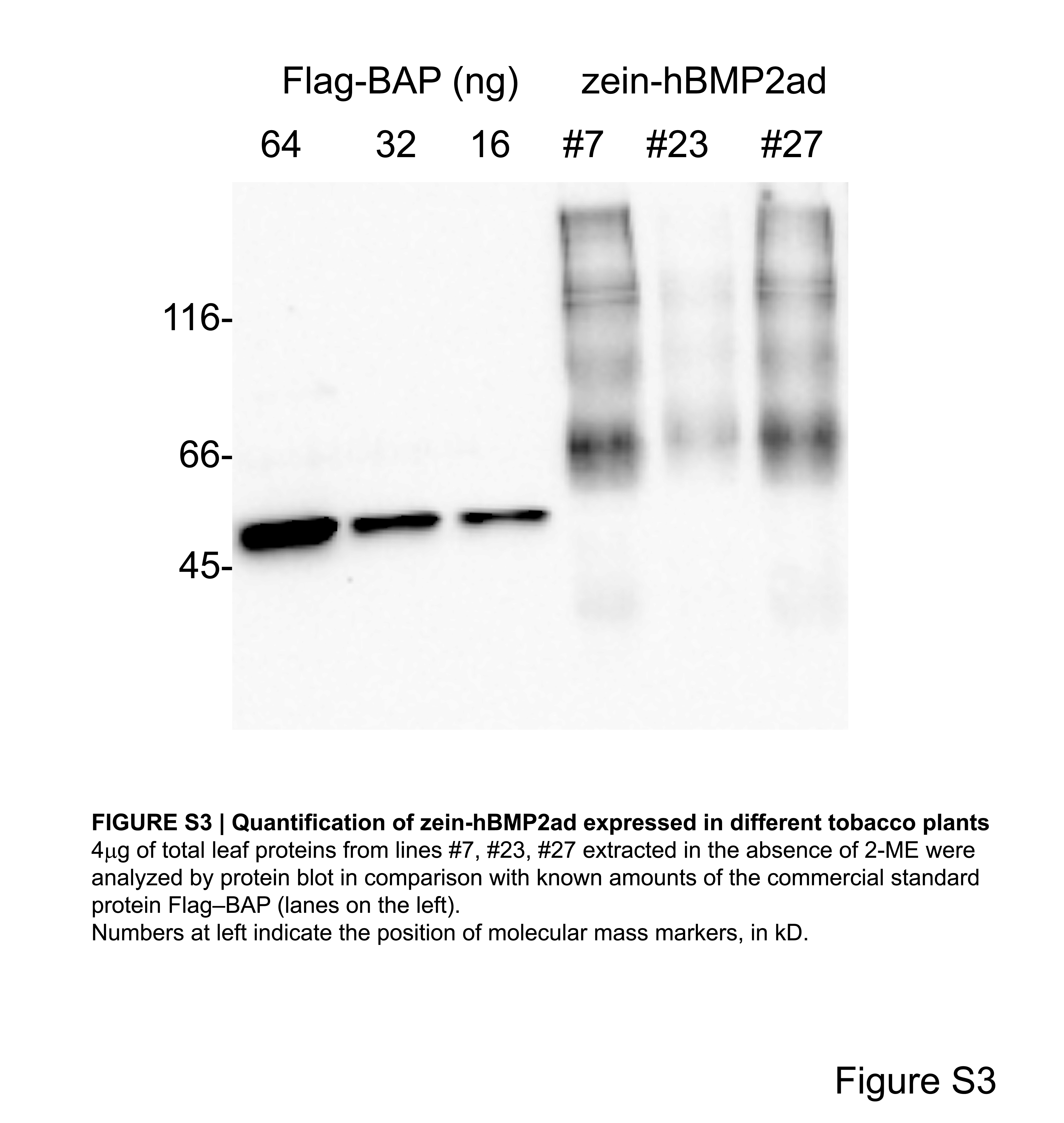

Supplement: Supplementary file 3 [file Image3.JPEG]
